# First person – Kate Ding

**DOI:** 10.1242/dmm.053103

**Published:** 2026-09-01

## Abstract

First Person is a series of interviews with the first authors of a selection of papers published in Disease Models & Mechanisms, helping researchers promote themselves alongside their papers. Kate Ding is first author on ‘ Gut-derived Unpaired-3 cytokine signaling promotes systemic hypoxia tolerance in *Drosophila*’, published in DMM. Kate conducted the research described in this article while a research assistant in Dr Savraj Grewal's lab at University of Calgary, Calgary, Canada, and is now a Molecular Technician at Applied Spatial Omics Centre, University of Calgary, investigating how spatial organization in tissues and organs influences cellular responses to stress and disease.

**Figure:**
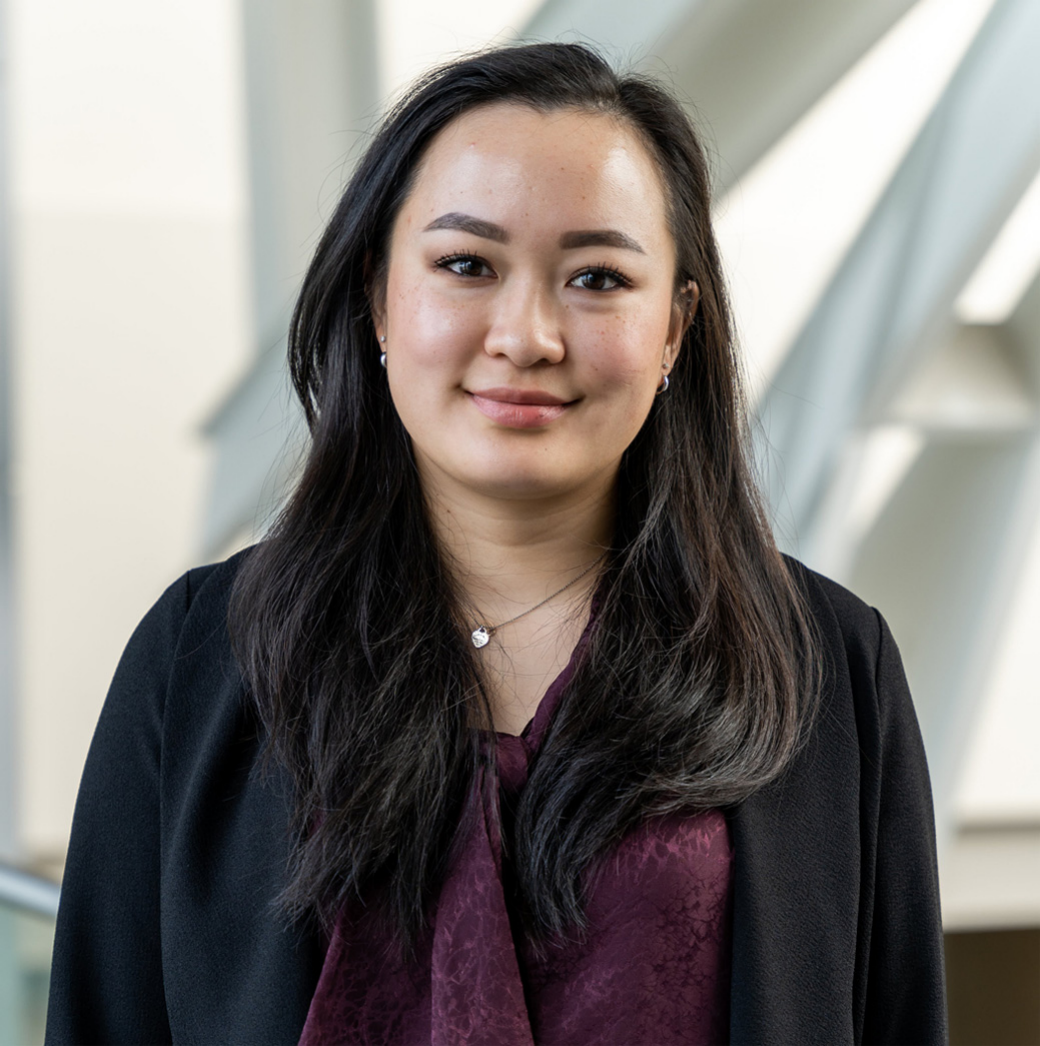
Kate Ding

**Who or what inspired you to become a scientist?**

I've always been curious about how the natural world works, especially biology, so science was my favourite subject in school. Like many students starting university, I wasn't sure what career I wanted until I joined a research lab as a summer student after my first year. That experience showed me what research was really about: asking questions, troubleshooting experiments, reading the literature and working with other scientists to solve problems. I continued seeking out research opportunities throughout my degree because I loved the challenge of discovering something new. What continues to motivate me is being part of a community that's driven by curiosity and by the hope that a better understanding of biology can, ultimately, improve human health.

What continues to motivate me is being part of a community that's driven by curiosity and by the hope that a better understanding of biology can, ultimately, improve human health

**What is the main question or challenge in disease biology you are addressing in this paper? How did you go about investigating your question or challenge?**

The main challenge addressed in this study is understanding how different organs communicate with one another to help an entire organism survive when oxygen levels are low, a condition known as systemic hypoxia. While scientists know a great deal about how individual cells respond to low oxygen, much less is known about how whole-body responses are coordinated. Using the fruit fly *Drosophila* as a model organism, we investigated whether the cytokine Upd3, a relative of the human inflammatory molecule IL-6, plays a role in this process. We exposed flies to low-oxygen conditions and measured changes in gene activity, survival and signalling pathways. We used genetic approaches to remove or reduce Upd3 and related signalling components in specific tissues to identify where the signal originates and where it acts. We also examined how another major hypoxia regulator, HIF-1α (Sima), influences this pathway. Together, these experiments allowed us to define a gut-to-fat body communication network that promotes survival during systemic hypoxia.

**How would you explain the main findings of your paper to non-scientific family and friends?**

We discovered that when oxygen levels drop, the intestine sends out an emergency signal that helps the rest of the body cope with the stress. This signal is especially important for female flies, helping them survive in low-oxygen environments. The message travels from the gut to other organs involved in managing energy and body resources, which then activate protective programs that improve survival. We also found that the amount of this signal must be carefully controlled: too little is harmful, but too much is also dangerous. Another oxygen-sensing system acts like a brake to prevent the signal from becoming excessive. In other words, survival during low oxygen depends not only on detecting the problem but also on coordinating communication between organs and keeping that communication balanced.

… this work may help identify new therapeutic strategies aimed at improving the body's ability to tolerate low oxygen while avoiding harmful inflammatory responses

**What are the potential implications of these results for disease biology and the possible impact on patients?**

Many human diseases – including sleep apnoea, chronic lung disease, COVID-19, stroke and other respiratory disorders – involve periods of reduced oxygen supply. Our findings suggest that molecules related to Upd3, such as the human cytokine IL-6, may have beneficial roles in helping the body adapt to low oxygen, in addition to their well-known roles in inflammation. The study also highlights the importance of communication between organs during stress and suggests that both insufficient and excessive cytokine signalling can be harmful. Understanding how these signals are regulated could help explain why inflammatory responses become damaging in some hypoxia-related diseases. In the long term, this work may help identify new therapeutic strategies aimed at improving the body's ability to tolerate low oxygen while avoiding harmful inflammatory responses. Although the work was performed in fruit flies, many of the signalling pathways involved are evolutionarily conserved and may provide insights relevant to human health.

**Figure:**
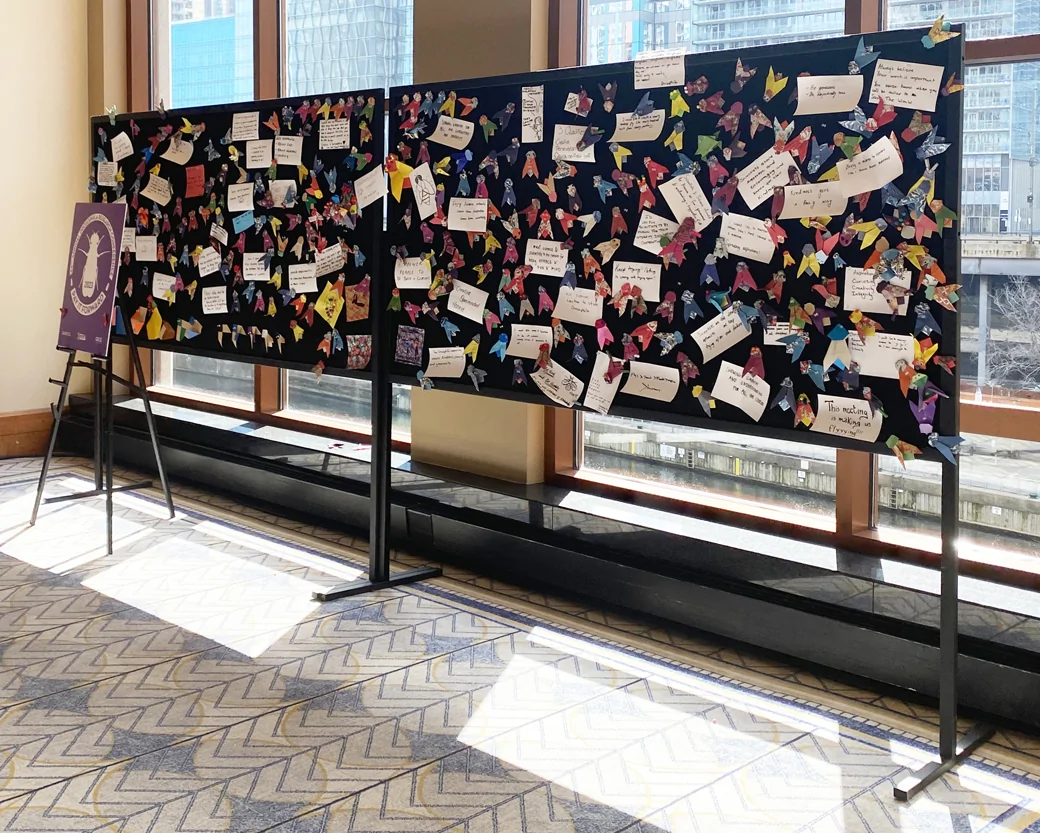
The origami fly station at Dros23, Chicago, IL, USA, which I helped organize.

**Why did you choose DMM for your paper?**

We chose DMM because our study addresses a fundamental question in disease biology: how the whole body adapts to low oxygen. Although our work uses *Drosophila*, the signalling pathways we studied are highly conserved and have clear relevance to human diseases associated with hypoxia. DMM has a strong track record of publishing research that bridges basic biology and disease mechanisms, making it an ideal home for our work.

**Given your current role, what challenges do you face and what changes could improve the professional lives of other scientists in this role?**

One of the biggest challenges facing early-career scientists is balancing a passion for research with long-term career and financial stability. Many talented researchers leave academia not because they lose interest in science, but because of limited opportunities and job security. Creating more stable career paths and increasing support for research staff would help retain experienced scientists and strengthen the research community as a whole.

**What's next for you?**

I recently joined the Applied Spatial Omics Centre at the University of Calgary, where I'm working with cutting-edge spatial omics technologies. I'm excited to learn how these approaches can reveal how cells interact within tissues and how spatial organization influences disease. Ultimately, I'd like to apply these technologies to better understand complex disease processes and improve how we study human health.

**Tell us something interesting about yourself that wouldn't be on your CV**

I love travelling and trying local food wherever I go. Exploring new places is one of my favourite ways to learn about different cultures.

**What do you want to see next in the field?**

One question that emerged from our study is why female flies require this signalling pathway to survive low oxygen whereas males do not. I'm excited to see more work exploring sex differences in stress responses, as these are increasingly recognized as important in many diseases. *Drosophila* offers powerful genetic tools to dissect these mechanisms, and I hope our findings encourage others to investigate how biological sex influences adaptation to stress.

## References

[DMM053103C1] Ding, K., Bodkhe, P., Lee, B., Ingelson-Filpula, W. A., Polan, D., Wycislik, A., Cheung, T., Wu, S., Rahim, R. and Grewal, S. S. (2026). Gut-derived Unpaired-3 cytokine signaling promotes systemic hypoxia tolerance in *Drosophila*. *Dis. Model. Mech.* 19, dmm052999. 10.1242/dmm.052999 42464911 PMC13580592

